# Supplementary material for: Maturation of the human striatal dopamine system revealed by PET and quantitative MRI
Source: Nat Commun. 2020 Feb 12;11:846. doi: 10.1038/s41467-020-14693-3 (PMC7015913; doi:10.1038/s41467-020-14693-3)
Supplement: Supplementary file 1 — Supplementary Information [file 41467_2020_14693_MOESM1_ESM.pdf]

Maturation of the human striatal dopamine system revealed by PET and quantitative MRI

Laresen et al.

Supplementary Information.

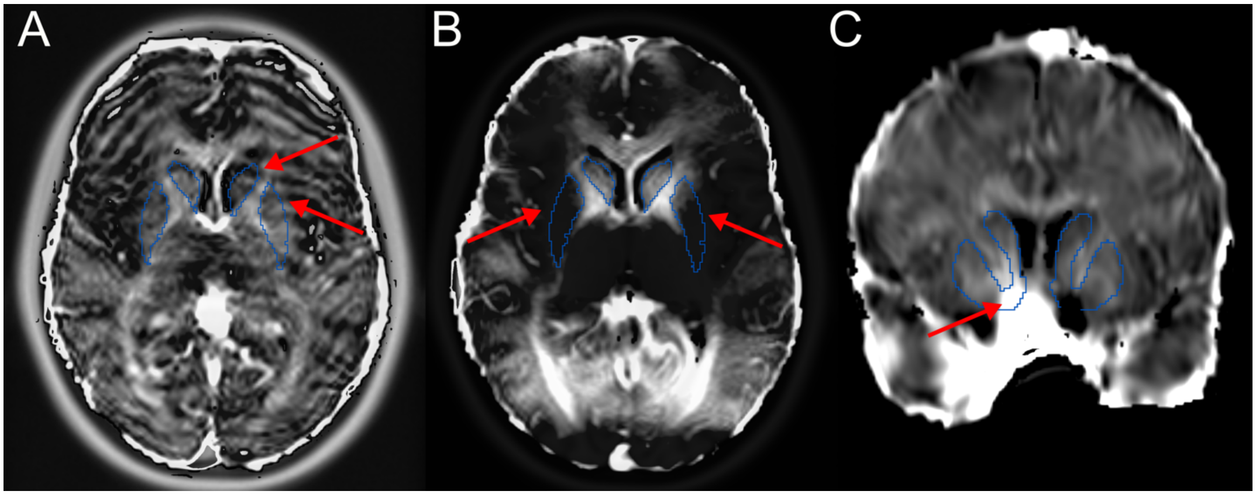

Supplementary Figure 1. Examples of artifacts present in R2’ data that were identified during quality assessment. All R2’ scans were assessed for data quality. Scans that contained artifacts related to (A) motion, (B) shimming, or (C) macroscopic field inhomogeneity that impacted the striatum were excluded from all analyses. The outline of the striatum is indicated in blue. The red arrows indicate examples of areas where artifacts impact striatum.

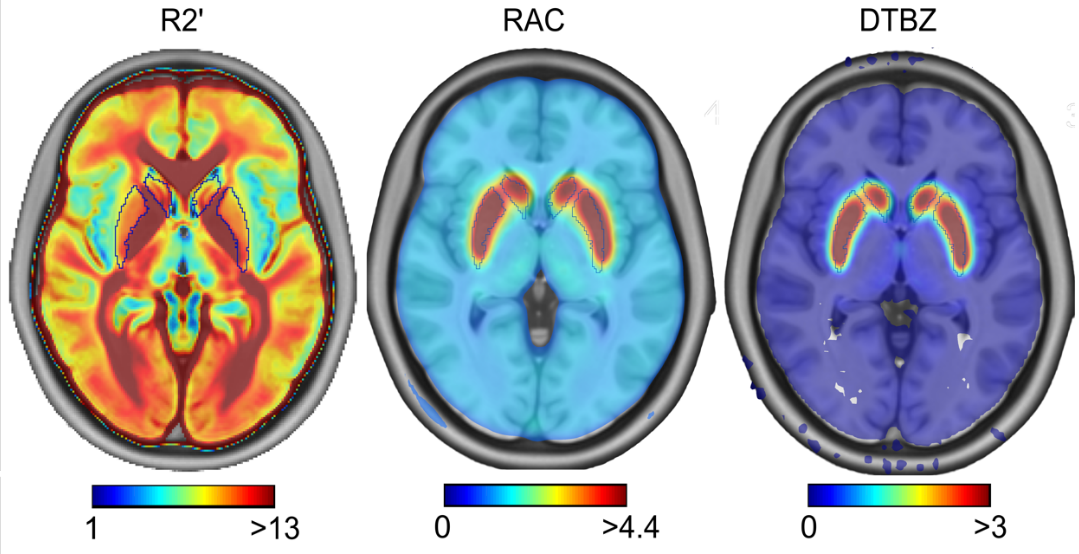

Supplementary Figure 2. Average images for all included R2’, [11C]Raclopride BP_{ND} (RAC), and [11C]Dihydrotetrabenazine BP_{ND} (DTBZ) sessions. Blue outline indicates the striatum.
