# Supplementary material for: Maturation of the human striatal dopamine system revealed by PET and quantitative MRI
Source: Nat Commun. 2020 Feb 12;11:846. doi: 10.1038/s41467-020-14693-3 (PMC7015913; doi:10.1038/s41467-020-14693-3)
Supplement: Supplementary file 3 — Reporting Summary [file 41467_2020_14693_MOESM3_ESM.pdf]

nature research

Corresponding author(s): Bart Larsen
Last updated by author(s): Jun 13, 2019

Reporting Summary

Nature Research wishes to improve the reproducibility of the work that we publish. This form provides structure for consistency and transparency in reporting. For further information on Nature Research policies, see Authors & Referees and the Editorial Policy Checklist .

Statistics

For all statistical analyses, confirm that the following items are present in the figure legend, table legend, main text, or Methods section.

n/a	Confirmed	
☐	☒	The exact sample size (*n*) for each experimental group/condition, given as a discrete number and unit of measurement
☐	☒	A statement on whether measurements were taken from distinct samples or whether the same sample was measured repeatedly
☐	☒	The statistical test(s) used AND whether they are one- or two-sided<br>*Only common tests should be described solely by name; describe more complex techniques in the Methods section.*
☐	☒	A description of all covariates tested
☐	☒	A description of any assumptions or corrections, such as tests of normality and adjustment for multiple comparisons
☐	☒	A full description of the statistical parameters including central tendency (e.g. means) or other basic estimates (e.g. regression coefficient) AND variation (e.g. standard deviation) or associated estimates of uncertainty (e.g. confidence intervals)
☐	☒	For null hypothesis testing, the test statistic (e.g. *F*, *t*, *r*) with confidence intervals, effect sizes, degrees of freedom and *P* value noted<br>*Give P values as exact values whenever suitable.*
☒	☐	For Bayesian analysis, information on the choice of priors and Markov chain Monte Carlo settings
☒	☐	For hierarchical and complex designs, identification of the appropriate level for tests and full reporting of outcomes
☐	☒	Estimates of effect sizes (e.g. Cohen's *d*, Pearson's *r*), indicating how they were calculated

Our web collection on statistics for biologists contains articles on many of the points above.

Software and code

Policy information about availability of computer code

Data collection	All imaging data was collected on a Siemens mMR dual modality PET/MR scanner.
Data analysis	Processing of PET data was done using PMOD v3.6 (PMOD Technologies LLC., Zürich, Switzerland) and FreeSurfer 5.3. Simplified Reference Tissue Model was implemented in MIAKAT. T1 images and R2/R2* images were processed in AFNI. All additional statistics were performed in MATLAB (2018B).

For manuscripts utilizing custom algorithms or software that are central to the research but not yet described in published literature, software must be made available to editors/reviewers. We strongly encourage code deposition in a community repository (e.g. GitHub). See the Nature Research guidelines for submitting code & software for further information.

Data

Policy information about availability of data

All manuscripts must include a data availability statement. This statement should provide the following information, where applicable:
- Accession codes, unique identifiers, or web links for publicly available datasets
- A list of figures that have associated raw data
- A description of any restrictions on data availability

The data that support the findings of this study are available from the corresponding author upon reasonable request.

Field-specific reporting

Please select the one below that is the best fit for your research. If you are not sure, read the appropriate sections before making your selection.

Life sciences study design

All studies must disclose on these points even when the disclosure is negative.

Sample size	The sample was selected to span the adolescent and early adulthood stages of development, and efforts were made to ensure comparable representation of each sex. The total sample size was selected based power estimates from prior developmental studies. All participants reported no history of psychiatric or neurological disorder, and all participants provided informed consent.
Data exclusions	A total of 56 sessions were excluded from R2' analyses, 21 sessions were excluded from DTBZ analyses, and 12 sessions were excluded from RAC analyses. Exclusions were made based on poor data quality as described in the manuscript.
Replication	Based on the unique features of the dataset, there was no replication sample collected.
Randomization	The sample was a random community driven sample based on study volunteers. There were no experimental groups.
Blinding	All researchers were blind to subject characteristics and identity during all stages of data processing and quality control.

Reporting for specific materials, systems and methods

We require information from authors about some types of materials, experimental systems and methods used in many studies. Here, indicate whether each material, system or method listed is relevant to your study. If you are not sure if a list item applies to your research, read the appropriate section before selecting a response.

Materials & experimental systems

n/a	Involved in the study	
☒	☐	Antibodies
☒	☐	Eukaryotic cell lines
☒	☐	Palaeontology
☒	☐	Animals and other organisms
☐	☒	Human research participants
☒	☐	Clinical data

Methods

n/a	Involved in the study	
☒	☐	ChIP-seq
☒	☐	Flow cytometry
☒	☐	MRI-based neuroimaging

Human research participants

Policy information about studies involving human research participants

Population characteristics	One hundred forty-six adolescents and young adults participated in the study (ages 12-30; M = 19.56, SD = 5.01; 71 males, 75 females). Ninety-two participants also participated in a follow-up experimental session that occurred approximately 18 months (M = 18.76, SD = 0.135) after the first visit (n = 146 unique participants, 238 total sessions).
Recruitment	Participants were recruited from the community based on flyers and other advertisements. All participants volunteered to participate.
Ethics oversight	All experimental procedures in this study complied with the Code of Ethics of the World Medical Association (1964 Declaration of Helsinki) and the Institutional Review Board at the University of Pittsburgh.

Note that full information on the approval of the study protocol must also be provided in the manuscript.
